# Sertraline and inflammatory markers in major depression: a systematic review and meta-analysis

**DOI:** 10.1186/s12991-025-00596-4

**Published:** 2025-09-29

**Authors:** ZiLiang Xie, Zheng Gao, Xue Li, Shuo Li, Fan Tang, Caiyi Zhang

**Affiliations:** 1 Department of Psychiatry, The Affiliated Xuzhou Oriental Hospital of Xuzhou Medical University, Xuzhou, 221000 China; 2 Department of Psychiatry, First Clinical College, Xuzhou Medical University, Xuzhou, 221000 China; 3 Department of Psychiatry, Second Clinical College, Xuzhou Medical University, Xuzhou, 221000 China; 4 The Key Lab of Psychiatry, Xuzhou Medical University, Xuzhou, 221000 China

**Keywords:** Sertraline inflammatory markers MDD systematic review and meta-analysis

## Abstract

**Background:**

Increasing evidence suggests a link between major depressive disorder (MDD) and inflammatory pathways. Sertraline, a commonly used selective serotonin reuptake inhibitor (SSRI), may influence inflammatory cytokine levels. This systematic review and meta-analysis aimed to elucidate the association between the antidepressant sertraline and inflammatory processes and to delineate the roles of key cytokines within this context.

**Methods:**

Electronic searches of the Web of Science, MEDLINE, and Embase databases (from inception to August 8, 2025) yielded 890 unique records. The included studies measured serum concentrations of inflammatory cytokines in patients with major depressive disorder (MDD) before and after sertraline treatment, with subsequent analysis of the effects of sertraline on these biomarkers. Two independent investigators performed the literature screening and data extraction. Pooled effect estimates were calculated using random effects meta-analysis models.

**Results:**

Eleven studies (406 participants) were included. The results demonstrated significant increases in both IL-6 levels (SMD = 0.87; 95% CI: 0.16 to 1.58; Z = 2.41; *p* = 0.02) and TNF-α levels (SMD = 0.76; 95% CI: 0.14 to 1.71; Z = 2.30; *p* = 0.02) following sertraline treatment.

**Conclusion:**

Sertraline significantly modulates IL-6/TNF-α levels, suggesting that this pathway may partly mediate its antidepressant effects.

**Supplementary Information:**

The online version contains supplementary material available at 10.1186/s12991-025-00596-4.

## Introduction

Major depressive disorder (MDD) is a debilitating mood disorder characterized by persistent low mood, diminished interest or pleasure (anhedonia), and loss of pleasure and is often accompanied by symptoms such as impaired concentration, appetite changes, sleep disturbances, and feelings of worthlessness [1]. The global burden of MDD is substantial and increasing. According to the World Health Organization (WHO), the prevalence of MDD increased by 28% during the COVID-19 pandemic [2]. Affecting more than 300 million individuals worldwide, MDD is projected to become the leading global source of disease burden by 2030 [3]. Currently, the main treatment for depression remains pharmacotherapy [4]. Sertraline, a widely prescribed selective serotonin reuptake inhibitor (SSRI), is a first-line agent for treating MDD and several other psychiatric conditions [5]. Despite its extensive clinical use, the precise mechanisms underlying its antidepressant effects have not been fully elucidated.

Emerging preclinical and clinical research has indicated that sertraline may partially exert its antidepressant effects through immunomodulation [6, 7]. Previous studies have shown that sertraline may alleviate depressive symptoms by affecting inflammatory pathways [8, 9]. Its mechanisms of action may involve inhibiting the NF-κB pathway, blocking IκBα degradation, and reducing the nuclear translocation of NF-κB, thereby downregulating the expression of proinflammatory genes and alleviating neuronal damage caused by inflammatory factors (e.g., TNF-α, IL-6, and IL-1β) [10, 11]. Some studies have suggested that sertraline may regulate the tryptophan metabolic pathway and suppress IDO activation induced by proinflammatory factors (e.g., IFN-γ and TNF-α) [12, 13], thus restoring the function of the serotonergic system. Other studies have indicated that sertraline can act on the TLR4/NF-κB pathway in microglia, inhibit the assembly of the NLRP3 inflammasome, and reduce the maturation and release of IL-1β [14–16]. Despite extensive evidence on the immunomodulatory role of antidepressants, no prior meta-analysis has focused specifically on the effects of sertraline on the levels of TNF-α and IL-6.

Sertraline, an SSRI widely used clinically in the treatment of depression, has a half-life of 26 h and has been approved by the FDA for the treatment of various psychiatric disorders, including major depressive disorder (MDD), obsessive-compulsive disorder (OCD), panic disorder, posttraumatic stress disorder (PTSD), and social anxiety disorder [17]. This study used a meta-analytic approach to explore the effects of sertraline use on the levels of inflammatory factors (particularly IL-6 and TNF-α) in clinical settings. We hypothesized that sertraline reduces the levels of circulating IL-6 and TNF-α in MDD patients.

## Methods

This systematic review and meta-analysis adhered to the PRISMA reporting guidelines [18] and was designed in accordance with methodological standards from the Cochrane Handbook for Systematic Reviews of Interventions [19]. The study protocol was prospectively registered with PROSPERO (CRD42024507002).

### Search strategy

Systematic searches were conducted in PubMed, Embase, and Web of Science from database inception through August 8, 2025. The search strategy (Supplement 1) incorporated a combination of Medical Subject Headings (MeSH) and free-text terms, including “sertraline,” “major depressive disorder,” and “cytokines.” Two investigators independently performed literature screening, initially reviewing titles and abstracts, followed by full-text assessment of potentially relevant records. Discrepancies were resolved through consultation with a third investigator.

### Inclusion and exclusion criteria

This systematic review used the following PICOS-defined inclusion criteria:

Population: Patients meeting the diagnostic criteria for major depressive disorder per the DSM-IV/V or ICD-10/11.

Intervention: Sertraline monotherapy.

Comparison: Placebo controls, active comparators, or no-treatment controls.

Outcomes: Serum cytokine concentration changes (primary endpoints: TNF-α and IL-6) before versus after treatment.

Study Designs: Randomized controlled trials (RCTs), healthy-control studies, case‒control studies, or placebo-controlled trials.

Studies were excluded if they met any of the following criteria: (1) review articles, case reports, conference abstracts, animal studies, or in vitro experiments; (2) duplicate publications; (3) data from polypharmacy regimens; (4) populations not meeting diagnostic criteria for major depressive disorder; (5) significant comorbidities, including pregnancy, renal impairment, malignancies, or uncontrolled systemic conditions; or (6) incomplete data reporting.

### Data extraction

Key data extracted included first author, publication year, age, continent, country, city, hospital, sample size, sex distribution, treatment duration, medication dosage, assessed factors and their measurement methods, and study design. The corresponding outcomes were recorded in Tables 1 and 2. The quality of each included study was evaluated using the Cochrane risk of bias assessment tool.

**Table 1:** Social-demographic characteristics

Author/Year/Country	Age(year)	Continent	Country	City	Hospital	Number of participants (Male/Female)
Xiang et al [25]/2023/China	12~17	Asia	China	Chongqing	The First Affiliated Hospital of Chongqing Medical University	61（15/46）
Taraz et al[31]/2020/Iran	60±22	Asia	Iran	Tehran	Taleghani Hospital	21(12/9)
Sutcigil et al[30]/2007/Turkey	34.78 ± 7.42	Asia	Turkey	Istanbul	Gülhane Military Medical Academy.	23（12/11）
Simon et al[28]/2021/Germany	18~60	Europe	Germany	Munich	Medical faculty of Ludwig Maximilians University	19
Liu et al[27]/2024/China	16.5±2.45	Asia	China	WuHan	Wuhan Mental Health Center, Wuhan Hospital	65（23/42）
Rawdin et al[23]/2013/America	37±10.77	North America	America	California	University of California, San Francisco (UCSF)	17
Abbasi et al[24]/2012/Iran	34.2 ± 6.90	Asia	Iran	Tehran	Roozbeh psychiatric hospital	20 (14/6)
Abbasian et al[29]/2022/Iran	34.60±9.86	Asia	Iran	Tehran	Roozbeh Psychiatric Hospital	15
Majmasanaye et al[22]/2024/Iran	34.60±9.86	Asia	Iran	Tehran	Hospital of Iran University of Medical Sciences	33（7/26）
Min et al[32]/2025/China	15(14,16)	Asia	China	Lishui	The Second People’s Hospital of Lishui	50（17/33）
Wu et al[26]/2025/China	13.66 ± 2.56	Asia	China	Wenzhou	Wenzhou Medical University Affiliated Fifth Hospital	82

Age values shown as mean ± SD, range, or median (IQR). Gender distribution specified where available; studies without gender notation had unreported demographic composition. Geographical locations reflect recruitment centers. Abbreviations: SD = standard deviation; IQR = interquartile range

**Table 2:** Characteristics and key findings of included studies on cytokine changes in MDD patients treated with sertraline

Author/Year/Country	Number of participants (n)	Treatment time	Dosage (mg/d)	Evaluation Method	Controlled and uncontrolled/randomized	Cytocines	Results
Xiang et al[25]/2023/China	61	8 weeks	25~200	Serum（ELISA）	Healthy control	IL6、TNF-α	Serum IL-6 remained significantly elevated in MDD vs. HC both pre- and post-treatment
Taraz et al[31]/2013/Iran	21	12 weeks	50 ~ 100	Serum（ELISA）	Randomized, double-blind	IL6、TNF-α	After 12 weeks of treatment in the sertraline group, the serum TNF-α levels decreased.
Sutcigil et al[30]/2007/Turkey	23	8 weeks	50 ~ 100	Serum（ELISA）	Healthy controls	TNF-α	The decrease in TNF-α after treatment was not significantly related to sertraline treatment
Simon et al[28]/2021/Germany	19	6 weeks	50 ~ 100	Serum（ELISA）	Randomized, double-blind, placebo-controll	TNF-α	There were no significant differences in baseline TNF-α levels
Liu et al[27]/2024/China	65	4 weeks	25 ~ 50	Serum（ELISA）	Randomized, double-blind, placebo-controll	IL6、TNF-α	TNF-α/IL-6 decreased significantly in combination vs. monotherapy groups post-treatment
Rawdin et al[23]/2013/America	17	8 weeks	50 ~ 200	Serum（ELISA）	Healthy control	IL6	Serum IL-6 increased significantly in MDD post-8-week sertraline treatment
Abbasi et al[24]/2012/Iran	20	6 weeks	100 ~ 200	Serum（ELISA）	Randomized double-blind	IL6	Sertraline reduced serum IL-6 vs. celecoxib but had lower response and remission rates
Abbasian et al[29]/2022/Iran	15	6 weeks	25 ~ 100	Serum（ELISA）	randomized, double-blind,	IL6、TNF-α	Combo therapy significantly decreased biomarkers vs. monotherapy (NS)
Majmasanaye et al[22]/2024/Iran	33	8 weeks	＞50	Serum（ELISA）	double-blind	IL6	IL-6 levels showed no significant changes.
Min et al[32]/2025/China	50	8 weeks	25~200	Serum（ELISA）	retrospective analysis	IL6、TNF-α	The sertraline group demonstrated more pronounced changes in IL-6 and TNF-α levels
Wu et al[26]/2025/China	82	4 weeks	25~200	Serum（ELISA）	randomized, double-blind	IL6、TNF-α	Sertraline significantly decreased IL-6/TNF-α levels with sustained TNF-α reduction

ELISA = enzyme-linked immunosorbent assay; HC = healthy controls; MDD = major depressive disorder

### Statistical analyses

Statistical analysis was performed using RevMan 5.4.1 software. The standardized mean difference (SMD) with corresponding 95% confidence intervals (CIs) was calculated to evaluate treatment effects through pre-post comparisons. The standardized mean difference (SMD) was calculated using the formula: SMD = (Mean₁ - Mean₂)/SDₚₒₗₑd. In this study, paired-data standardized mean difference (SMD) calculations were performed in accordance with the Cochrane methodological standards for longitudinal outcome synthesis [19]. The Cochrane risk of bias assessment tool for nonrandomized studies (RoBANS) was used to evaluate the risk of bias. Six domains were assessed: (1) participant selection, (2) confounding variables, (3) exposure measurement, (4) outcome assessment blinding, (5) outcome data completeness, and (6) reporting selectivity. The authors provided scoring guidelines for each domain, with assessments categorized as low, unclear, or high risk of bias [20]. Study heterogeneity was assessed using the I² statistic. Given the substantial heterogeneity observed across studies, subgroup analyses and sensitivity analyses were conducted to validate the selection of a random-effects model over a fixed-effects model for meta-analysis [21].

## Results

### Study selection and characteristics

**Fig. 1:**
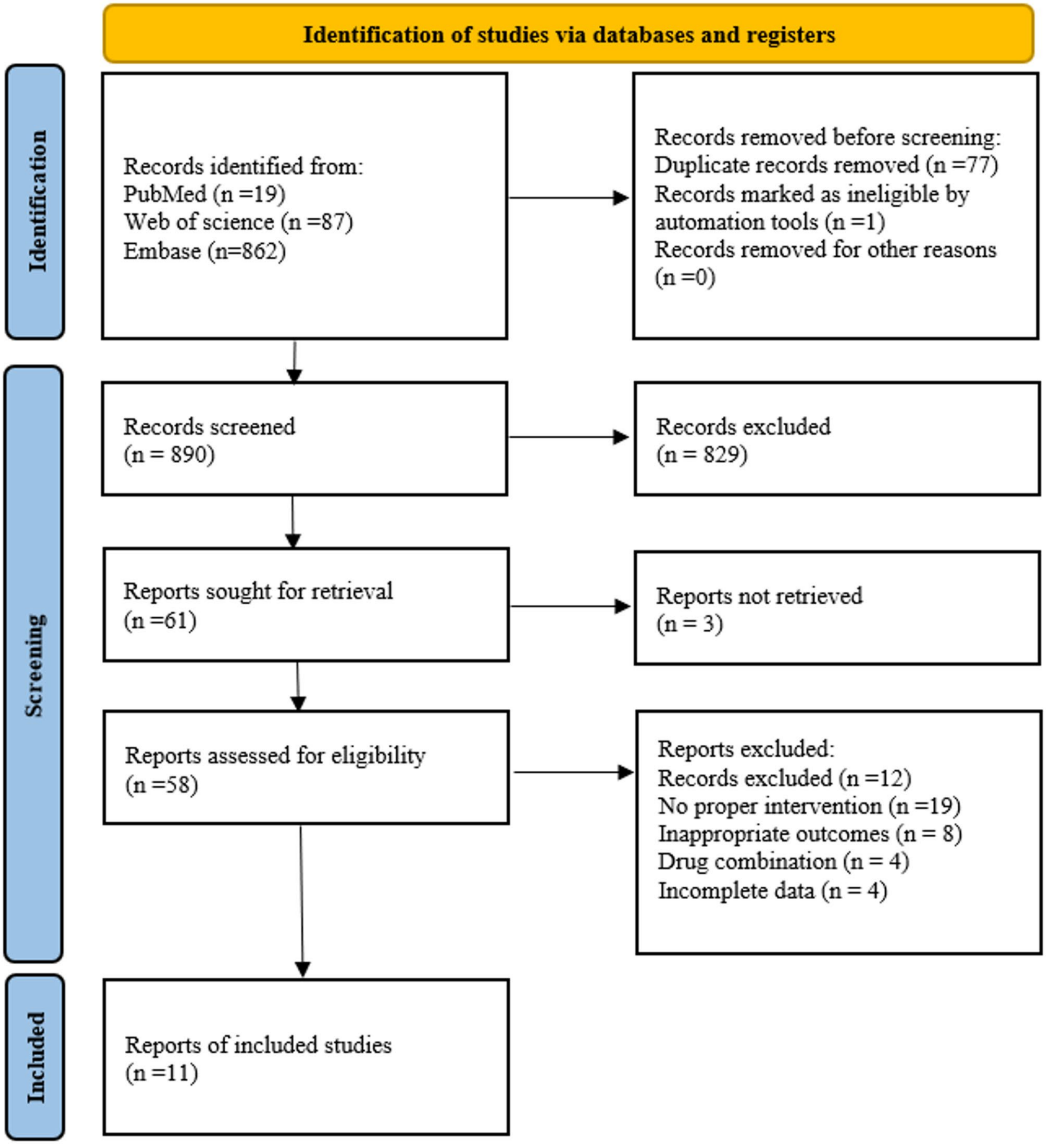
Meta-Analyses Flow Diagram

A total of 890 records were identified through initial electronic database searches. Following title/abstract screening using reference management software, 829 records were excluded because they did not meet the eligibility criteria. After the full-text assessment, 50 records were excluded, yielding 11 studies [22–32] for final inclusion in the meta-analysis. The study selection process is illustrated in Fig. 1.

### Interleukin-6

**Fig. 2:**
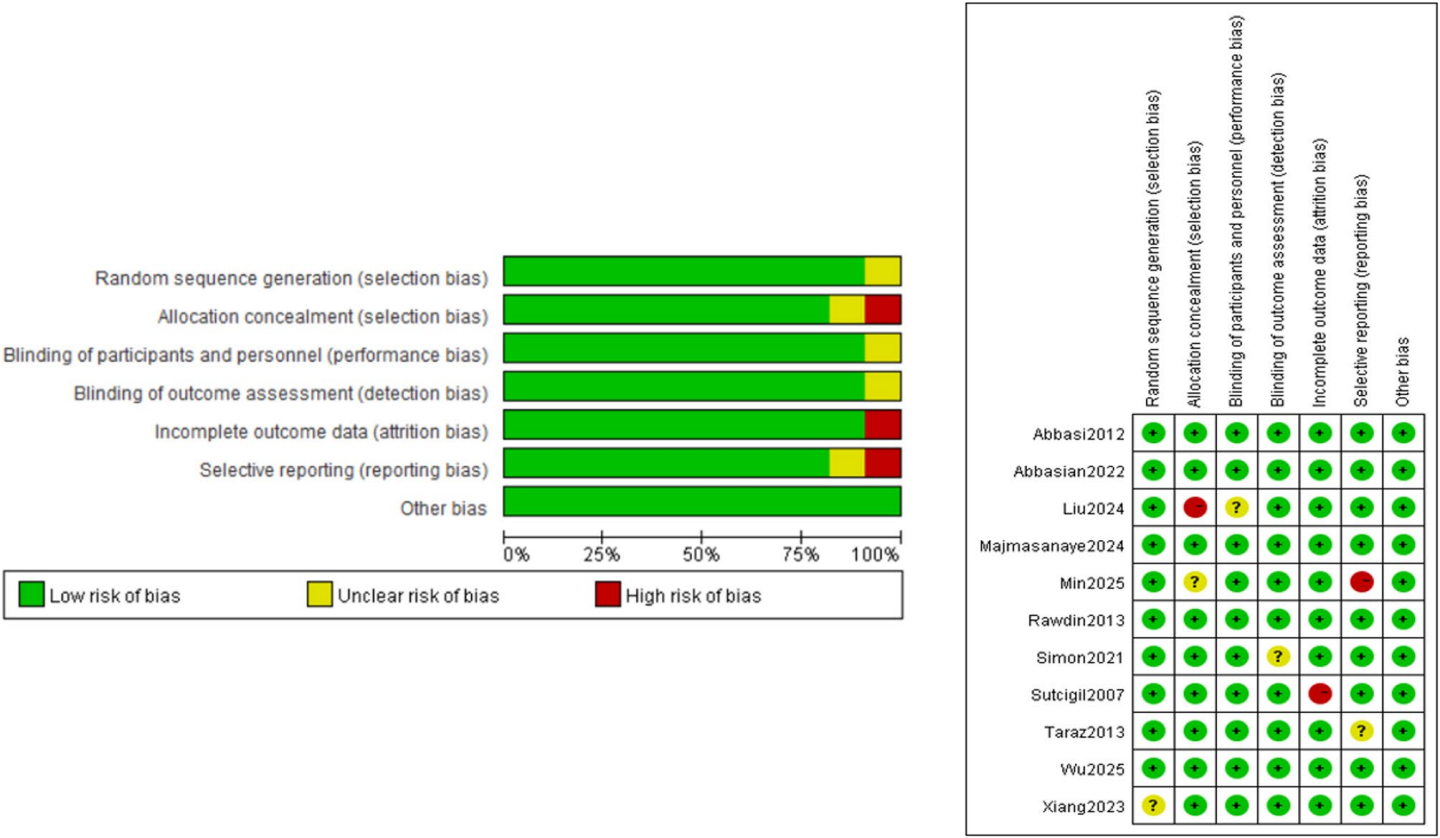
Effects of Sertraline on IL-6 and TNF-α Levels; Effect sizes (SMD) are indicated by solid squares with horizontal error bars representing 95% confidence intervals; Solid diamonds denote the pooled mean effect size; (A) IL-6; (B) TNF-α

**Fig. 3:**
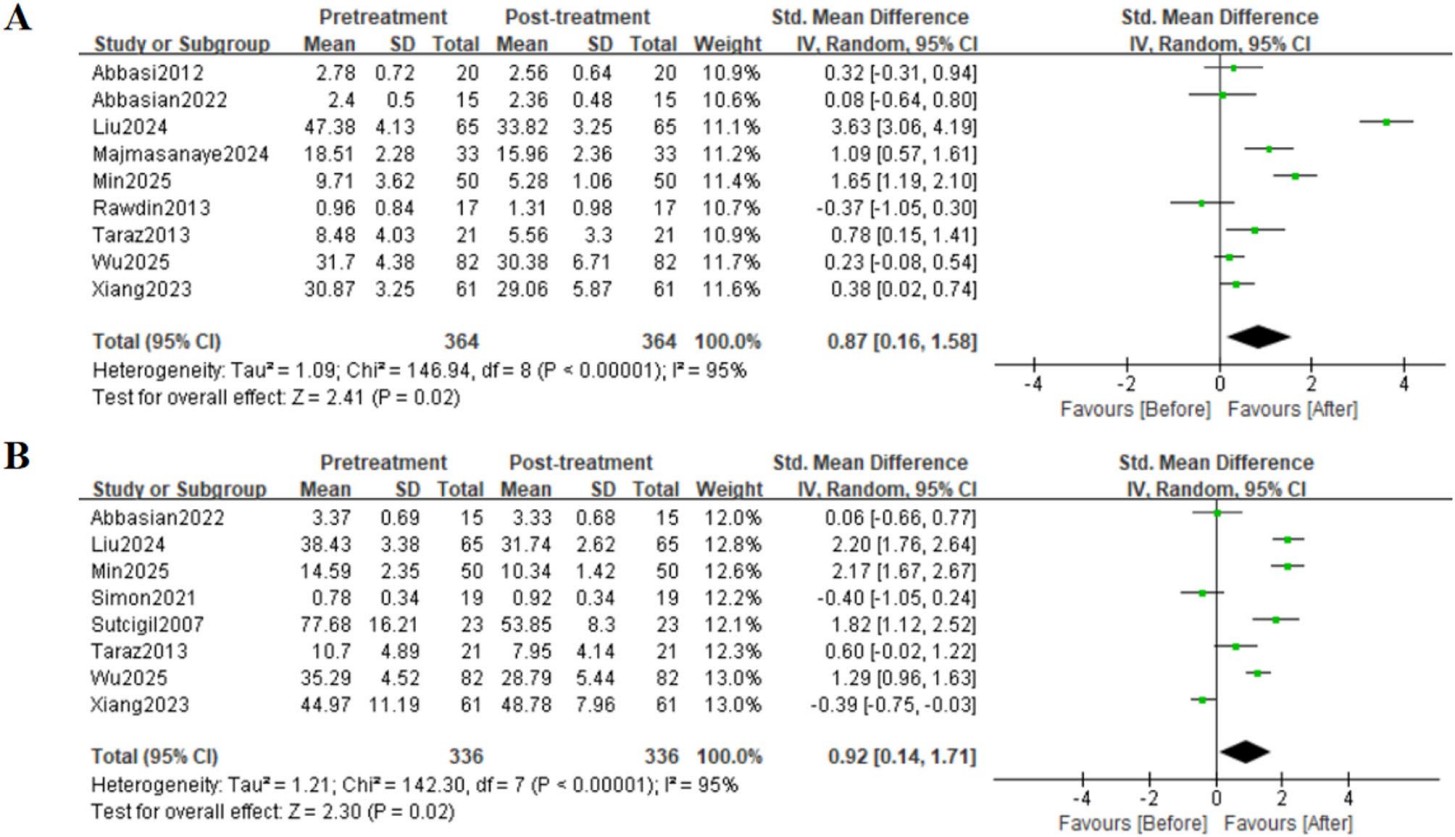
Funnel plot for comparison of effect sizes of IL-6 and TNF-α before and after sertraline treatment; black circles indicate the effect size of each study; (A) IL-6; (B) TNF-α

This meta-analysis included 9 studies on the effect of sertraline on IL-6 levels (Figs. 2A and 3A). Analysis based on a random-effects model revealed that sertraline treatment had a significant effect on serum IL-6 levels in patients (SMD = 0.87, 95%CI: 0.16 to 1.58; Z = 2.41, *p* = 0.02). There was significant heterogeneity among the studies (τ²=1.09, χ²=146.94, df = 8, *p* < 0.00001, I²=95%).

### Tumour necrosis factor-alpha

Among the included studies, 8 evaluated TNF-α levels (Figs. 2B and 3B). Analysis using a random-effects model revealed that sertraline intervention had a significant effect on TNF-α levels (SMD = 0.76, 95%CI: 0.14 to 1.71; Z = 2.30, *p* = 0.02). There was significant heterogeneity across the studies (τ²=1.21, χ²=142.30, df = 7, *p* < 0.00001, I²=95%).

### Risk of bias (RoB)

**Fig. 4:**
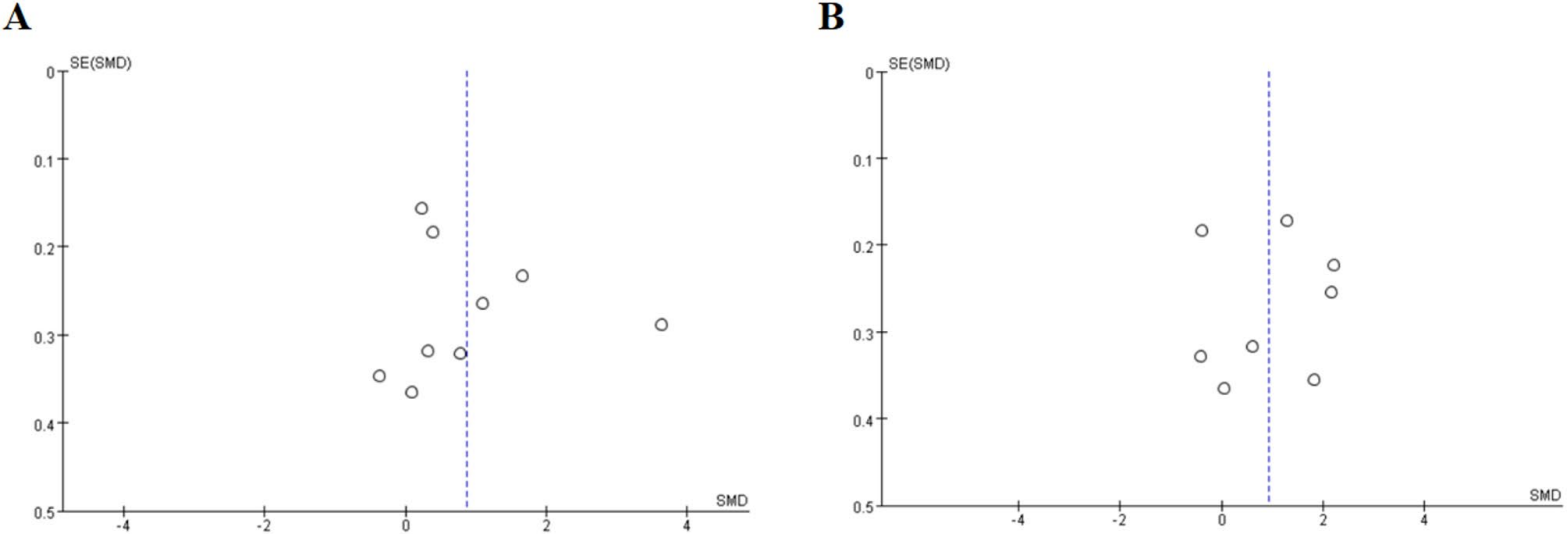
Quality assessment using Cochrane risk of bias tool; Green indicates low risk; yellow indicates unknown risk; red indicates high risk

The quality assessment of each study was conducted by 2 independent authors (LZ, ZG, and SL) in accordance with the Cochrane Handbook for Systematic Reviewers. Among the included studies, 3 were assessed as having a high risk of bias, and 3 were assessed as having some concerns. A summary of the quality assessment is presented in Fig. 4.

### Subgroup analysis

#### Age-stratified subgroup analysis

**Fig. 5:**
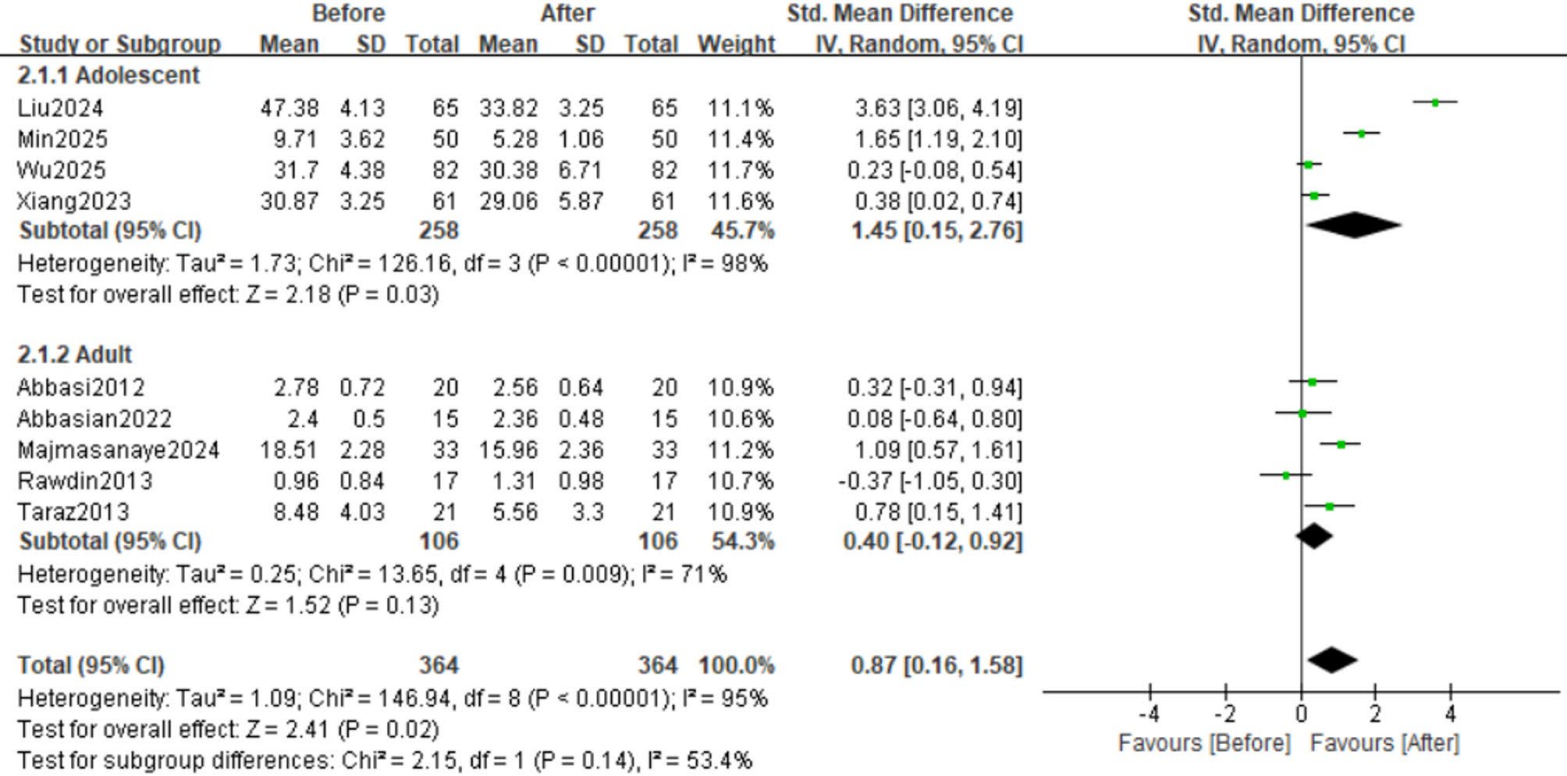
Forest plot of IL-6 levels between adolescent and adult subgroups; effect sizes (SMDs) are indicated by solid squares with horizontal error bars representing 95% confidence intervals; solid diamonds denote the pooled mean effect size

**Fig. 6:**
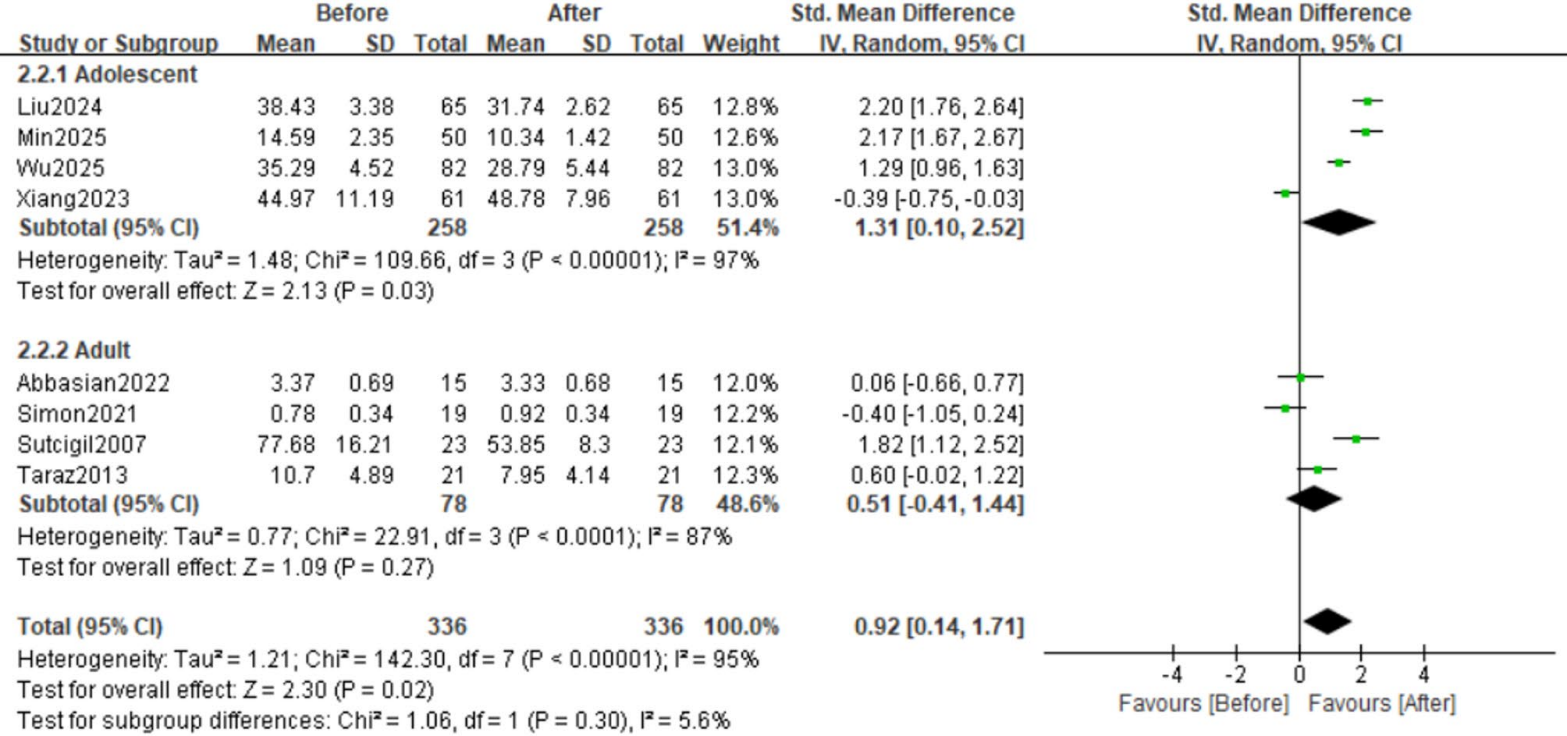
Funnel Plot of effect sizes for IL-6/TNF-α in adolescent vs. adult Subgroups; Red hollow squares represent effect sizes for adults, and black circles represent effect sizes for adolescents; (A) IL-6; (B) TNF-α

We conducted subgroup analyses by age, categorizing participants aged 12–18 years as adolescents and those > 18 years as adults. Four studies reported changes in IL-6 levels in the adolescent subgroup, whereas five studies reported changes in the adult subgroup. Compared with the overall pooled analysis, the analysis revealed lower heterogeneity in the adult subgroup (SMD = 0.40, 95%CI: −0.12 to 0.92, Z = 1.52, *p* = 0.13; τ²=0.25, χ²=13.65, df = 4, *p* = 0.009, I²=71%), whereas substantially higher heterogeneity was observed in the adolescent subgroup (SMD = 1.45, 95%CI: 0.15 to 22.76, Z = 2.18, *p* = 0.03; τ²=1.73, χ²=126.16, df = 3, *p*<0.00001, I²=98%). This differential heterogeneity may have affected the robustness of the findings (Figs. 5 and 6A).

**Fig. 7:**
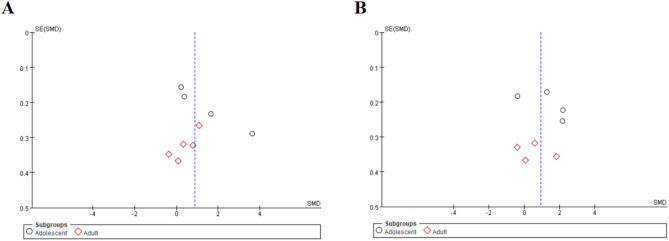
Forest plot of TNF-α levels between adolescent and adult subgroups; effect sizes (SMDs) are indicated by solid squares with horizontal error bars representing 95% confidence intervals; solid diamonds denote the pooled mean effect size

Changes in TNF-α levels paralleled those in IL-6 levels in both age subgroups. Four studies evaluated TNF-α level changes in the adolescent subgroup (SMD = 1.31, 95% CI: 0.10 to 2.52; Z = 2.13, *p* = 0.03; τ² = 1.48, χ² = 109.66, df = 3, *p* < 0.00001, I² = 97%). Similarly, four studies assessed TNF-α changes in adults (SMD = 0.51, 95% CI: −0.41 to 1.44; Z = 1.09, *p* = 0.27; τ² = 1.21, χ² = 142.30, df = 7, *p* < 0.00001, I² = 87%)(Figs. 7 and 6B).

#### Subgroup analysis by treatment duration

**Fig. 8:**
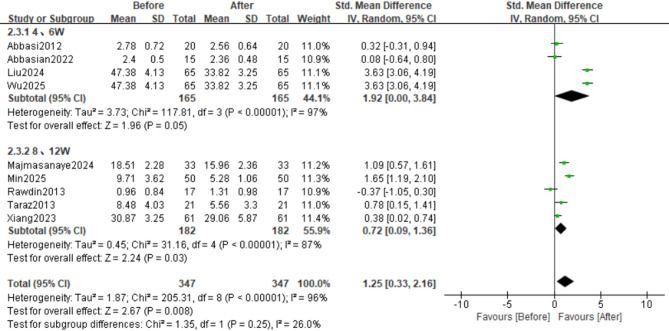
Forest Plot of IL-6 Levels between Short-term (4–6 week) and long-term (8–12 week) subgroups; Effect sizes (SMD) are indicated by solid squares with horizontal error bars representing 95% confidence intervals; Solid diamonds denote the pooled mean effect size

**Fig. 9:**
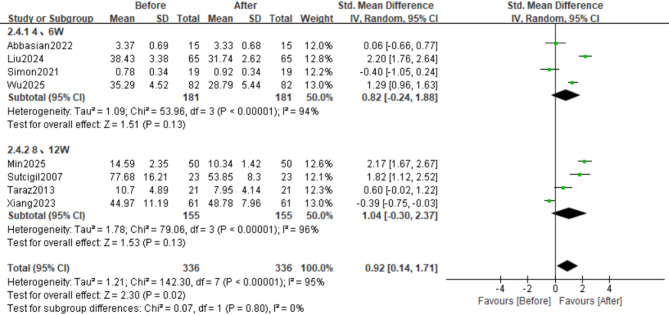
Funnel Plot of effect sizes for IL-6/TNF-α in short-term (4–6 week) vs. long-term (8–12 week) Subgroups; Red hollow squares represent treatment duration of 8–12 weeks, and black circles represent treatment duration of 4–6 weeks; (A) IL-6; (B) TNF-α

We conducted subgroup analyses stratified by treatment duration, defining participants who received 4–6 weeks of therapy as the short-term subgroup and those who received 8–12 weeks of therapy as the long-term subgroup. Four studies reported changes in IL-6 levels in the short-term therapy subgroup, whereas five studies reported changes in the long-term subgroup. Compared with the overall pooled analysis, the long-term subgroup analysis exhibited lower heterogeneity (SMD = 0.72, 95% CI: 0.09 to 1.36; Z = 2.24, *p* = 0.03; τ² = 0.45, χ² = 31.16, df = 4, *p* < 0.00001, I² = 87%). Conversely, heterogeneity remained unmitigated in the short-term subgroup (SMD = 1.92, 95% CI: 0.00 to 3.84; Z = 1.96, *p* = 0.05; τ² = 3.73, χ² = 117.81, df = 3, *p* < 0.00001, I² = 97%) (Figs. 8 and 9A).

**Fig. 10:**
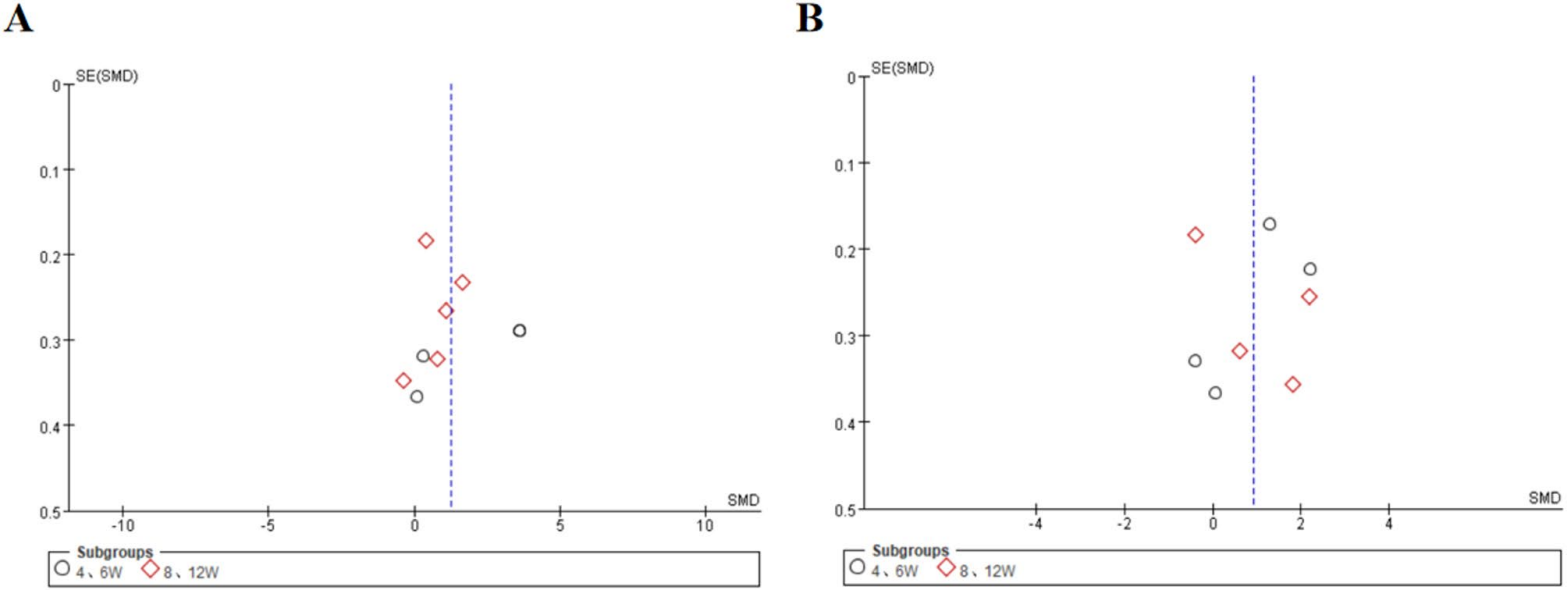
Forest Plot of TNF-α Levels between Short-term (4–6 week) and long-term (8–12 week) subgroups; Effect sizes (SMD) are indicated by solid squares with horizontal error bars representing 95% confidence intervals; Solid diamonds denote the pooled mean effect size

In contrast to changes in the levels of IL-6, changes in the levels of TNF-α were not significantly altered in either subgroup. In the short-term therapy group (*n* = 4 studies), we observed nonsignificant effects (SMD = 0.82, 95% CI: −0.24 to 1.88; Z = 1.51, *p* = 0.13; τ² = 1.09, χ² = 53.96, df = 3, *p* < 0.00001, I² = 94%). Similarly, the long-term therapy subgroup (*n* = 4 studies) demonstrated comparable nonsignificant outcomes (SMD = 1.04, 95% CI: −0.30 to 2.37; Z = 1.53, *p* = 0.13; τ² = 1.78, χ² = 79.06, df = 3, *p* < 0.00001, I² = 96%) despite substantial heterogeneity (Figs. 10 and 9B).

## Discussion

Many studies have indicated that the mechanism of action of sertraline in the treatment of depression is associated with inflammatory cytokines; however, its specific mechanism remains unclear. Our findings suggest that sertraline treatment can significantly affect the levels of IL-6 and TNF-α in patients with depression. These findings indicate that sertraline may, to a certain extent, alleviate depressive symptoms by regulating the levels of inflammatory cytokines.

This study confirmed that the levels of IL-6 and TNF-α in patients with depression significantly decreased before and after sertraline monotherapy. This finding is consistent with the results of some previous studies [33]. The reason for this phenomenon may be that sertraline inhibits the phosphorylation of IKKβ kinase, prevents it from degrading the NF-κB inhibitory protein IκBα, maintains the stability of IκBα, and thus inhibits the transcription of IL-6 and TNF-α by preventing the nuclear translocation of NF-κB [34]. Alternatively, sertraline may reduce the expression levels of IL-6 and TNF-α by activating HDAC to deacetylate p65, thereby preventing the nuclear translocation of NF-κB [35].

The results revealed differences in the quality of the included literature. Overall, most studies had a low risk of bias; however, the high risk of bias in some studies cannot be ruled out, and potential hidden bias may have exerted a certain effect on the results. Our findings indicated high heterogeneity among the studies, which may be attributed to differences in sample size, treatment duration, or age. This is a relatively common issue in meta-analyses [36]. To identify the sources of heterogeneity, we conducted subgroup analyses. Subgroup analysis stratified by age revealed that heterogeneity was somewhat reduced in the adult subgroup, while no significant reduction was observed in the adolescent subgroup. This may be because adolescents are in a critical period of growth and development, and their immune systems and neuroendocrine systems (such as the hypothalamic–pituitary–adrenal [HPA] axis) are not yet fully mature, leading to more unstable inflammatory regulatory mechanisms (e.g., influenced by pubertal hormone fluctuations and stages of brain development) [37, 38]. In addition, we performed a subgroup analysis stratified by treatment duration, and the results revealed no significant reduction in heterogeneity. This may be because the maximum treatment duration among the included studies was only 12 weeks, and the changes in inflammatory factors during this period may not follow a simple linear relationship [39]; therefore, the results should be interpreted with caution.

We performed a leave-one-out sensitivity analysis on the results, which indicated that the level of heterogeneity did not decrease significantly when any single study was excluded. These findings suggest that the results of our meta-analysis are reliable [40].

In addition, our findings may be subject to publication bias, as some studies had small sample sizes and some were non-RCT studies, both of which may contribute to publication bias [41]. Despite systematic searches across multiple databases and clinical trial registration platforms, publication bias may still exist. Such bias could stem from the selective publication of favourable data in pharmaceutical company-sponsored trials or a reduction in effect size after trim-and-fill correction, although it remains within the clinically significant range [42].

## Limitations

This study used meta-analysis to conduct a pooled analysis of changes in the levels of IL-6 and TNF-α before and after sertraline monotherapy. However, the study has several limitations: (1) A small number of studies were included, with a small total sample size, which may pose a risk of Type II error in statistical methods. (2) The analysed studies included nonrandomized controlled studies, which may increase the degree of heterogeneity to some extent. (3) The levels of inflammatory factors are influenced by multiple factors, and their changes in vivo do not have a simple linear relationship. Although we conducted subgroup analyses on the results and critically interpreted and explained them, we cannot rule out the impact of other confounding factors, such as genetics and diet. Therefore, whether sertraline alleviates depressive symptoms by regulating IL-6 and TNF-α levels still requires verification by more high-quality clinical studies and systematic reviews.

## Conclusion

Our study revealed that sertraline can regulate the levels of inflammatory factors to a certain extent and may alleviate clinical symptoms by reducing the levels of IL-6 and TNF-α. Additional well-designed studies are still needed in the future to verify these conclusions.

## Supplementary Information

Supplementary Material 1

## Data Availability

No datasets were generated or analysed during the current study.
